# The Spectrin-Actin-Based Periodic Cytoskeleton as a Conserved Nanoscale Scaffold and Ruler of the Neural Stem Cell Lineage

**DOI:** 10.1016/j.celrep.2018.07.005

**Published:** 2018-08-07

**Authors:** Meghan Hauser, Rui Yan, Wan Li, Nicole A. Repina, David V. Schaffer, Ke Xu

**Affiliations:** 1 Department of Chemistry, University of California, Berkeley, Berkeley, CA 94720, USA; 2 Department of Bioengineering, University of California, Berkeley, Berkeley, CA 94720, USA; 3 Department of Chemical and Biomolecular Engineering, University of California, Berkeley, Berkeley, CA 94720, USA; 4 Helen Wills Neuroscience Institute, University of California, Berkeley, Berkeley, CA 94720, USA; 5 Department of Molecular and Cell Biology, University of California, Berkeley, Berkeley, CA 94720, USA; 6 Division of Molecular Biophysics and Integrated Bioimaging, Lawrence Berkeley National Laboratory, Berkeley, CA 94720, USA; 7 Chan Zuckerberg Biohub, San Francisco, CA 94158, USA; 8 Lead Contact

## Abstract

Through three-dimensional STORM super-resolution microscopy, we resolve the spectrin-actin-based membrane cytoskeleton of neural stem cells (NSCs) and NSC-derived neurons, astrocytes, and oligodendrocytes. We show that undifferentiated NSCs are capable of forming patches of locally periodic, one-dimensional (1D) membrane cytoskeleton with ~180 nm periodicity. Such periodic structures become increasingly ordered and long-ranging as the NSCs mature into terminally differentiated neuronal and glial cell types, and, during this process, distinct 1D periodic ‘‘strips’’ dominate the flat 2D membranes. Moreover, we report remarkable alignment of the periodic cytoskeletons between abutting cells at axon-axon and axon-oligodendrocyte contacts and identify two adhesion molecules, neurofascin and L1CAM, as candidates to drive this nanoscale alignment. We thus show that a conserved 1D periodic membrane cytoskeletal motif serves as a nanoscale scaffold and ruler to mediate the physical interactions between cell types of the NSC lineage.

## In Brief

Hauser et al. employ three-dimensional STORM super-resolution microscopy to resolve the actin-spectrin-based membrane cytoskeleton in neural stem cells (NSCs) and NSC-derived neurons, astrocytes, and oligodendrocytes, revealing a highly conserved one-dimensional periodic cytoskeletal motif that serves as a nanoscale scaffold and ruler for intercellular interactions.

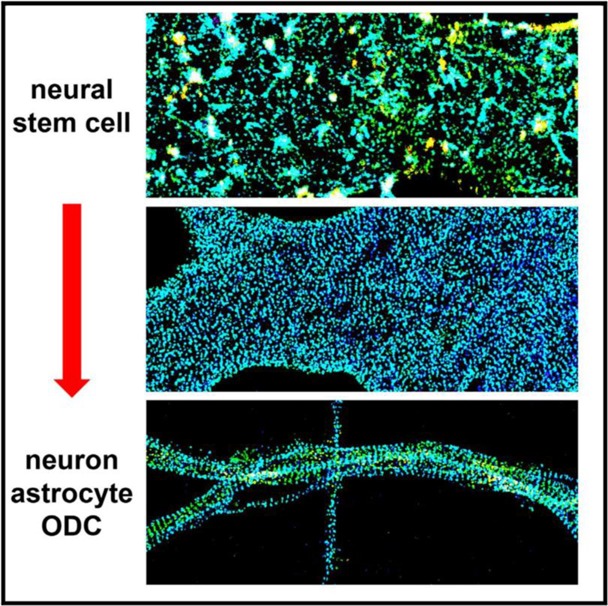

## INTRODUCTION

The recent discovery (Xu et al., 2013) of a highly structured and periodic membrane cytoskeleton in neurons via super-resolution microscopy (SRM) (Huang et al., 2010; Sahl et al., 2017) has kindled great interest in the ultrastructure of the membrane cytoskeleton in cells of the nervous system (Albrecht et al., 2016; Bär et al., 2016; D’Este et al., 2015, 2016, 2017; Ganguly et al., 2015; Han et al., 2017; He et al., 2016; Leite et al., 2016; Leterrier et al., 2015, 2017; Sidenstein et al., 2016; Xu et al., 2013; Zhong et al., 2014). Although initially noted in neuronal axons as adducin-capped actin rings connected by spectrin tetramers to form a periodic, one-dimensional (1D) lattice of well-defined, ~180- to 190-nm periodicity (Xu et al., 2013), related periodic or quasi-periodic cytoskeletal structures have also been observed in dendrites (D’Este et al., 2015; Han et al., 2017) and certain glial cell types (D’Este et al., 2016, 2017; He et al., 2016). Such periodic nanostructures are markedly different from the traditional view of the actin-based cytoskeleton in common mammalian cell types (e.g., dense filament networks and bundles in fibroblasts and epithelial cells) (Chhabra and Higgs, 2007; Pollard and Cooper, 2009; Xu et al., 2012) as well as the spectrin-actin-based cytoskeleton in erythrocytes (2D triangular lattices of short actin filaments connected by spectrin tetramers) (Baines, 2010; Bennett and Baines, 2001; Bennett and Gilligan, 1993; Fowler, 2013; Pan et al., 2018). Questions thus arise regarding what the common denominator is for cells that exhibit such 1D periodic arrangements, how such states are achieved during development, and which functions the highly conserved 180-to 190-nm periodicity may carry beyond the current discussions centered around axon initial segments (AISs) (Albrecht et al., 2016; Xu et al., 2013) and nodes of Ranvier (D’Este et al., 2017).

Although previous studies have examined the development of the periodic spectrin-actin cytoskeleton during the growth and/or regrowth of neurites for terminally differentiated neurons in dissociated hippocampal cultures (D’Este et al., 2015; Han et al., 2017; Xu et al., 2013; Zhong et al., 2014), *in vivo* neurons and supporting cells develop from stem cells (progenitors). For example, neural stem cells (NSCs) in the subgranular zone of the adult mammalian hippocampus can both proliferate with preserved multipotency and differentiate into all major cell types in the CNS, including neurons, astrocytes, and oligodendrocytes (Gage, 2000; Gage and Temple, 2013). Consequently, they play crucial roles in learning and memory and hold great potential for the treatment of neurological injuries and diseases.

Using three-dimensional stochastic optical reconstruction microscopy (3D-STORM) (Huang et al., 2008; Rust et al., 2006) SRM, here we resolved the membrane cytoskeleton in undifferentiated adult hippocampal NSCs as well as NSC-derived neurons, astrocytes, and oligodendrocytes. We found that undifferentiated NSCs are capable of forming patches of locally periodic membrane cytoskeletons of ~180- to 190-nm periodicity; these periodic structures become increasingly ordered and 1D as the NSCs differentiate into terminal cell types and that, during this process, distinct 1D periodic ‘‘strips’’ often dominate the flat 2D membranes. Moreover, we report remarkable structural alignment of the periodic membrane cytoskeleton between abutting cells at axon-axon and axon-oligodendrocyte contact sites and identify two adhesion molecules, neurofascin and L1CAM, as possible candidates to drive this alignment at the nanoscale. Together, our results indicate that a conserved 1D periodic membrane cytoskeleton motif serves as a nanoscale scaffold and ruler to mediate the interactions between different cell types of the NSC lineage.

## RESULTS

### The Actin, Spectrin, and Adducin Membrane Cytoskeleton of Undifferentiated NSCs Is Characterized by Patches of Periodic Patterns

**Figure 1.**
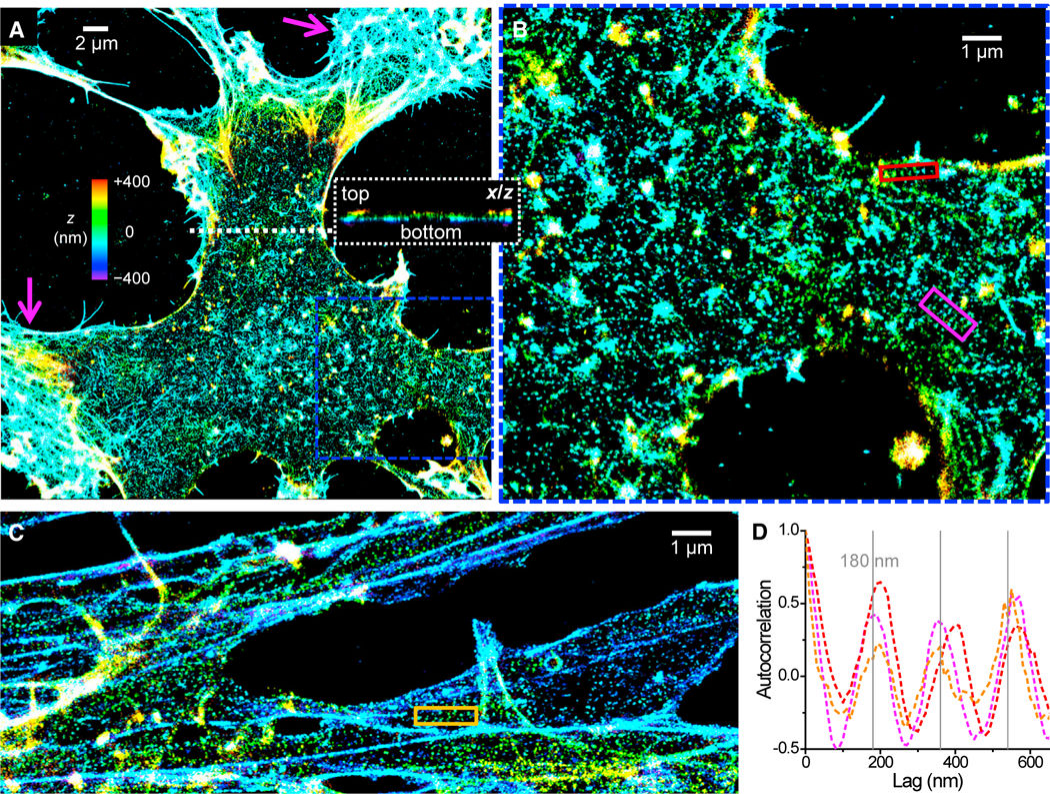
The Actin Cytoskeleton of Undifferentiated NSCs Contains Patches of Locally Periodic Patterns at the Nanoscale. (A)3D-STORM image of the phalloidin-labeled actin cytoskeleton of an undifferentiated NSC. Color represents height z (color bar; violet denotes closest to the substrate, and red denotes farthest away). Magenta arrows point to protruding edges. Inset: virtual cross-section of the 3D-STORM data in the xz plane along the white dotted line. (B) Enlargement of the blue box in (A). (C) Image of NSC processes. (D) One-dimensional autocorrelation along the red, magenta, and orange boxes in (B) and (C). Gray grid lines mark multiples of 180 nm. See also Figure S1.

Nestin-positive NSCs were isolated from adult rat hippocampi for *in vitro* culture (Peltier et al., 2010a, 2010b). 3D-STORM (Huang et al., 2008) was employed to resolve ultrastructures at ~25-nm spatial resolution. Figure 1 and Figure S1 show representative results of the phalloidin-labeled actin cytoskeleton of undifferentiated NSCs. Color was used to present the height (z) dimension. Because of the relatively shallow working depth range of 3D-STORM (~800 nm) (Huang et al., 2008), images often only captured the bottom (ventral) membrane cytoskeleton, whereas the top (dorsal) membrane cytoskeleton rapidly rose in height when away from cell edges and so disappeared from view (e.g., the cross-sectional view in the inset of Figure 1A).

Interestingly, although the protruding edges of the NSCs were dominated by dense actin networks (magenta arrows in Figure 1A), micrometer-sized patches of semi-regular lattice patterns of dot-like actin were observed across other parts of the cell membrane, interspersed with filament clusters and bundles (Figures 1A-1C; Figure S1). The observed dense networks and bundles are analogous to those found in common epithelial cells and fibroblasts (Chhabra and Higgs, 2007; Pollard and Cooper, 2009; Xu et al., 2012). The distinctive, dot-like lattice arrangement of actin we revealed, however, suggests an ultrastructure related to the spectrin-actin-based cytoskeleton of erythrocytes and neurons. For both systems, ~30-nm short actin filaments locate to junctional complexes connected by spectrin tetramers; however, a 2D polygonal lattice of ~80-nm junction-to-junction distance is found in erythrocytes (Baines, 2010; Bennett and Baines, 2001; Bennett and Gilligan, 1993; Fowler, 2013; Pan et al., 2018), whereas neuronal processes are often character-ized by 1D lattices of 180- to 190-nm periodicity (Albrecht et al., 2016; B är et al., 2016; D’Este et al., 2015, 2016, 2017; Ganguly et al., 2015; Han et al., 2017; He et al., 2016; Leite et al., 2016; Leterrier et al., 2015, 2017; Sidenstein et al., 2016; Xu et al., 2013; Zhong et al., 2014).

We found that the dot-like actin lattices in the NSCs contained both 2D polygonal and 1D linear arrangements (Figure 1; Figure S1). Notably, short (~1-μm) strips of local 1D periodicity were observed across the membrane (red, magenta, and orange boxes in Figure 1BC). 1D autocorrelations (Xu et al., 2013; Zhong et al., 2014) along the lengths of the strips yielded peaks at multiples of ~180–190 nm (Figure 1D), indicating a locally ordered 1D arrangement at this well-defined periodicity.

**Figure 2.**
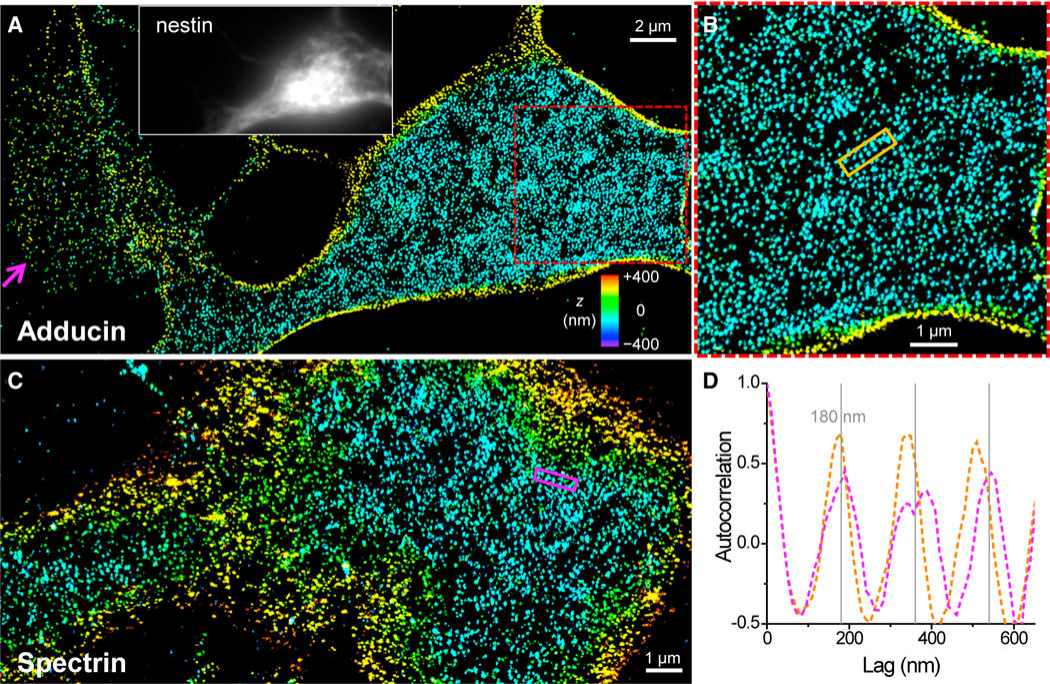
Adducin and Spectrin in Undifferentiated NSCs Are Characterized by Locally Periodic Patterns Commensurate with the Actin Lattices. (A) 3D-STORM image of immunolabeled adducin at the ventral (bottom) membrane of an undifferentiated NSC. The magenta arrow points to a protruding edge. Inset: immunofluorescence of the NSC marker nestin. (B) Enlargement of the red box in (A). (C) 3D-STORM image of immunolabeled βII spectrin (C terminus) at the ventral (bottom) membrane of an undifferentiated NSC. (D) One-dimensional autocorrelations along the orange and magenta boxes in (B) and (C). Gray grid lines mark multiples of 180 nm. See also Figure S1.

To determine whether the semi-ordered actin patches and strips in NSCs were indeed due to a spectrin-connected network as in erythrocytes and neurons, we performed 3D-STORM for immunolabeled adducin, a protein that caps short actin filaments and mediates their binding to spectrin (Baines, 2010; Bennett and Baines, 2001; Bennett and Gilligan, 1993; Matsuoka et al., 2000; Xu et al., 2013) as well as the C terminus of βII spectrin, which corresponds to the centers of spectrin tetramers (Baines, 2010; Bennett and Baines, 2001; Bennett and Gilligan, 1993; Xu et al., 2013). Patches of semi-ordered clusters were observed for both targets across the NSC membrane (Figure 2; Figure S1). Locally periodic 1D strips were again observed (orange and magenta boxes in Figure 2BC), for which 1D autocorrelations showed repeating peaks at multiples of ~180 nm (Figure 2D). Protruding edges were characterized by lower adducin labeling (magenta arrows in Figure 2A and Figure S1), suggesting a reduced presence of the spectrin-actin-based cytoskeleton. These results are commensurate with the actin ultrastructure we found (Figure 1; Figure S1).

Together, we revealed that, in undifferentiated NSCs, the membrane cytoskeleton in protruding edges is dominated by dense actin networks, similar to common cell types such as epithelial cells and fibroblasts, whereas, in the cell bodies and processes, actin, spectrin, and adducin assemble into patches of semi-ordered networks related to that of neurons and erythrocytes.

### The Membrane Cytoskeletons of NSC-Derived Mature Neurons, Astrocytes, and Oligodendrocytes Are Characterized by 1D Periodicity

We next examined NSCs that were differentiated into the terminal cell types of neurons, astrocytes, and oligodendrocytes by addition of retinoic acid and fetal bovine serum (Peltier et al., 2010a) for 10–14 days. Similar to the extensively characterized primary hippocampal neurons (D’Este et al., 2015; Ganguly et al., 2015; Han et al., 2017; Xu et al., 2013; Zhong et al., 2014), NSC-derived neurons exhibited a 1D periodic membrane cytoskeleton (Figure S2).

**Figure 3.**
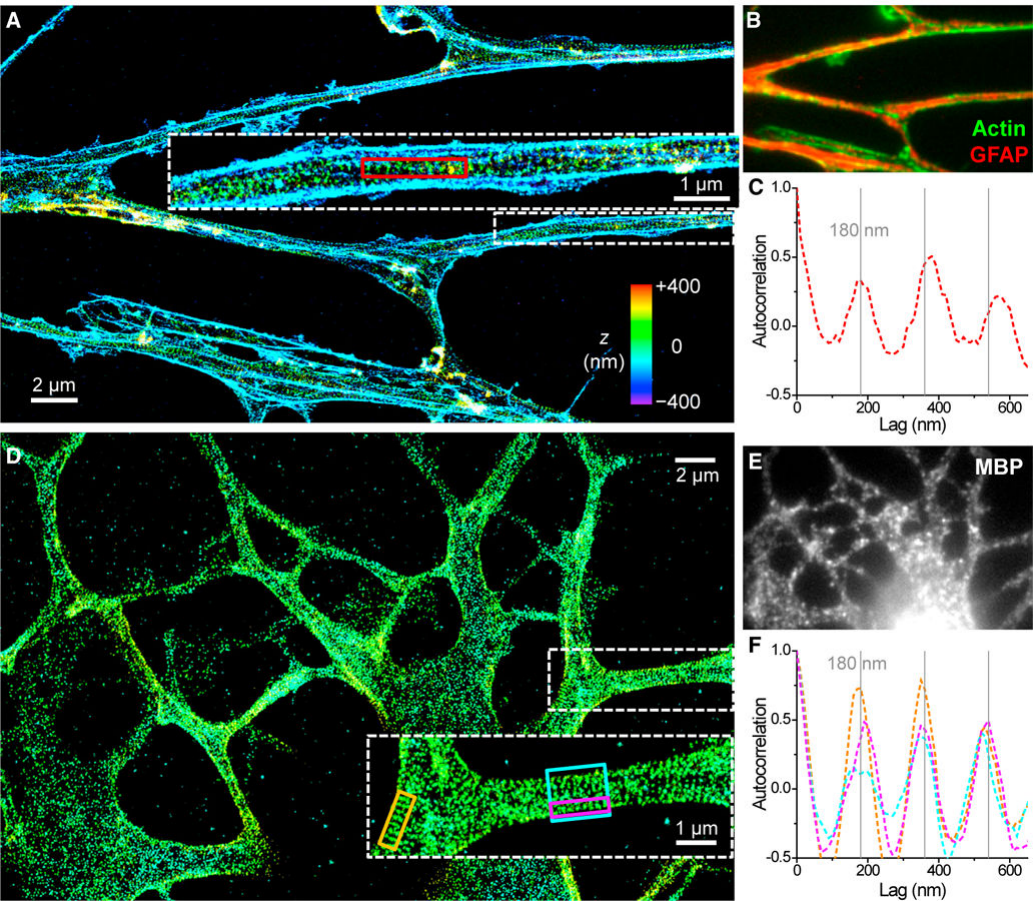
The Membrane Cytoskeletons of NSC-Derived Astrocytes and Oligodendrocytes Are Highly Periodic. (A) 3D-STORM image of the phalloidin-labeled actin cytoskeleton of an NSC-derived astrocyte. Inset: enlarged image of a process. (B) Overlapped epifluorescence of actin (green) and GFAP (red). (C) One-dimensional autocorrelation along the red box in (A). Gray grid lines mark multiples of 180 nm. (D) 3D-STORM image of immunolabeled adducin for an NSC-derived oligodendrocyte. Inset: enlarged image of a process. (E) MBP staining of the cell. (F) One-dimensional autocorrelations along the orange, magenta, and cyan boxes in (D). Gray grid lines mark multiples of 180 nm. See also Figures S2 and S3.

Interestingly, NSC-derived astrocytes, as identified by positive labeling of glial fibrillary acidic protein (GFAP) and negative labeling of nestin, also showed ordered 1D periodic patterns of actin (Figures 3A–3C) and spectrin (Figure S3A) in the often numerous processes originating from the same cell. We additionally obtained comparable results with primary astrocytes isolated from hippocampal tissues (Figure S3B). A recent study reported a partially periodic membrane cytoskeleton in primary astrocytes (He et al., 2016), whereas another study did not observe periodicity but noted possible technical causes (D’Este et al., 2016). Our results suggest that astrocytes are characterized by a ubiquitous periodic membrane cytoskeleton.

NSC-derived mature oligodendrocytes, identified by positive labeling of myelin basic protein (MBP), also exhibited an ordered 1D periodic membrane cytoskeleton of ~180 nm periodicity (see Figures 3D–3F for adducin and Figures S3C and S3D for spectrin). Here the processes were often wider in width (>1 μm) than in astrocytes and neurons and exhibited more frequent branching. 1D autocorrelation (Figure 3F) gave repeating peaks at multiples of ~180 nm for both local 1D strips on the membrane (orange and magenta boxes in Figure 3D) and the full width of processes (cyan box in Figure 3D). Cell bodies contained both 2D and 1D lattices (Figure 3D; Figure S3). Recent works report short-range actin-spectrin periodicity in oligodendrocyte precursor cells (NG2 glia) (D’Este et al., 2016; He et al., 2016). Our results show that MBP-positive, mature oligodendrocytes, meanwhile, are characterized by an extensive periodic membrane cytoskeleton.

### The 2D Membranes of Developing NSCs Are Characterized by Collages of 1D Strips of Periodic Membrane Cytoskeleton

**Figure 4.**
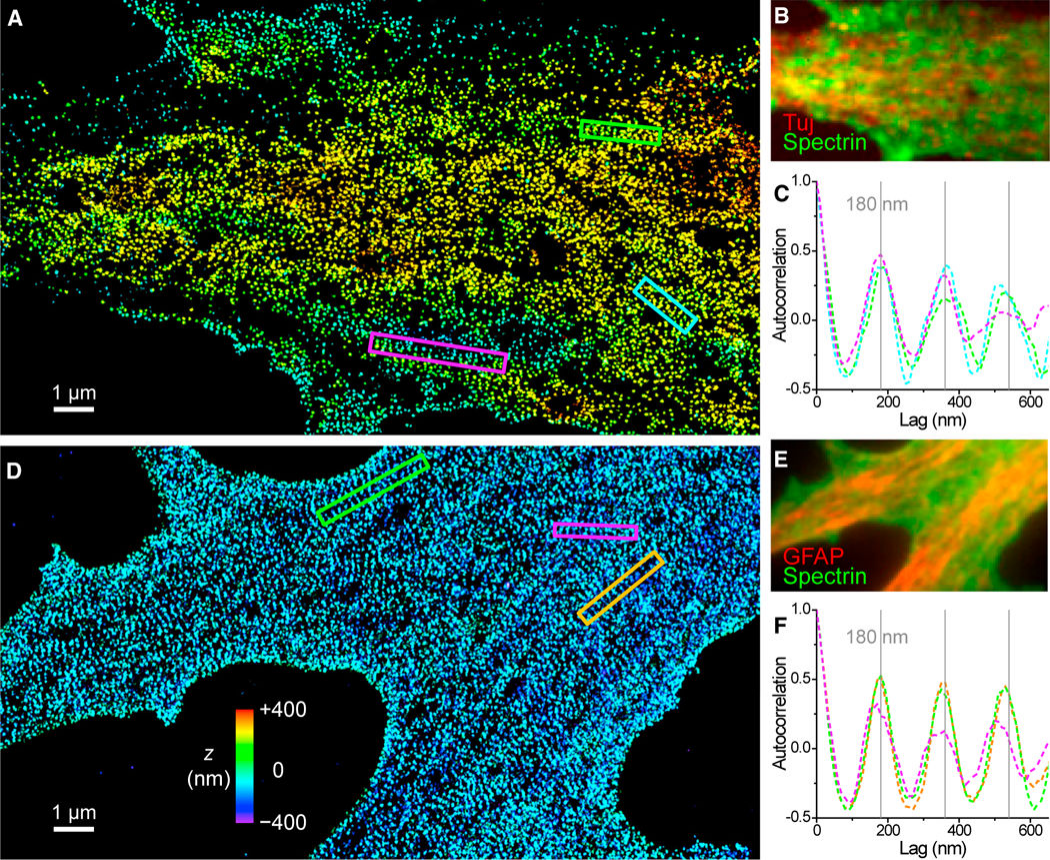
Collages of 1D Periodic Cytoskeletal Motifs on the 2D Membranes of Developing NSCs. (A) 3D-STORM image of βII spectrin (C terminus) at the dorsal (top) membrane of an NSC in transition to a neuron. (B) Overlapped epifluorescence of the neuron marker Tuj (neuron-specific class III β-tubulin; red) and βII spectrin (green) of the cell. (C) One-dimensional autocorrelations along the green, magenta, and cyan boxes in (A). Gray grid lines mark multiples of 180 nm. (D) 3D-STORM image of βII spectrin (C terminus) at the ventral (bottom) membrane of an NSC in transition to an astrocyte. (E) GFAP (red) and βII spectrin (green) staining of the cell. (F) One-dimensional autocorrelations along the green, magenta, and orange boxes in (D). Gray grid lines mark multiples of 180 nm. See also Figure S4.

Strikingly, we next found that, because the NSC is differentiated into terminal cell types, ordered cytoskeletal strips of 1D periodicity often dominated the flat 2D membranes; e.g., the cell bodies and the very wide, immature processes. Figure 4 presents 3D-STORM results of βII spectrin for two developing NSCs in their transitions to a neuron (Figures 4A–4C) and an astrocyte (Figures 4D–4F) after induction by retinoic acid and fetal bovine serum for 5 and 7 days, respectively. Strips of highly ordered 1D periodicity were observed across the 2D membranes. Each strip was 200–300 nm in width and up to ~4 μm in length, within which 1D periodicity was individually achieved (e.g., see the colored boxes in Figure 4AD). 1D correlations along the lengths of the strips showed highly ordered peaks at multiples of ~180 nm (Figure 4CF). These individual 1D periodic strips then collaged together with diverse orientations to cover the 2D membrane surface (Figure 4; see additional examples in Figure S4).

Collectively, our results indicate that the NSC has an innate capability to form a spectrin-actin-based periodic membrane cytoskeleton. Undifferentiated NSCs contain micrometer-sized actin-spectrin-adducin patches of local orderliness, and this ultrastructure becomes increasingly ordered and 1D over longer distances as the NSCs develop into neurons, astrocytes, and oligodendrocytes.

### The Conserved 1D Periodic Membrane Cytoskeletal Motif Is Aligned for Contacting Cells

Our results so far demonstrate a conserved 1D periodic membrane cytoskeletal ultrastructure that is inherited from the NSCs and shared by all NSC-derived cell types. It has been suggested that the spectrin-actin-based membrane cytoskeleton provides a mechanically flexible yet durable support for the neuronal membrane (Hammarlund et al., 2007; Xu et al., 2013), and SRM has shown that the periodic cytoskeleton organizes ion channels and structure-maintaining proteins at the AIS (Albrecht et al., 2016; D’Este et al., 2015; Leterrier et al., 2015; Xu et al., 2013). Although these functions may still be relevant for all cell types of the NSC lineage, the prevalence of the spectrin-actin-based cytoskeletal system across different NSC-derived cell types and its remarkable preference for a fixed 180- to 190-nm 1D periodicity prompted us to ask whether this conserved structural motif could act as a scaffold and/or ruler to control cell-cell interactions.

**Figure 5.**
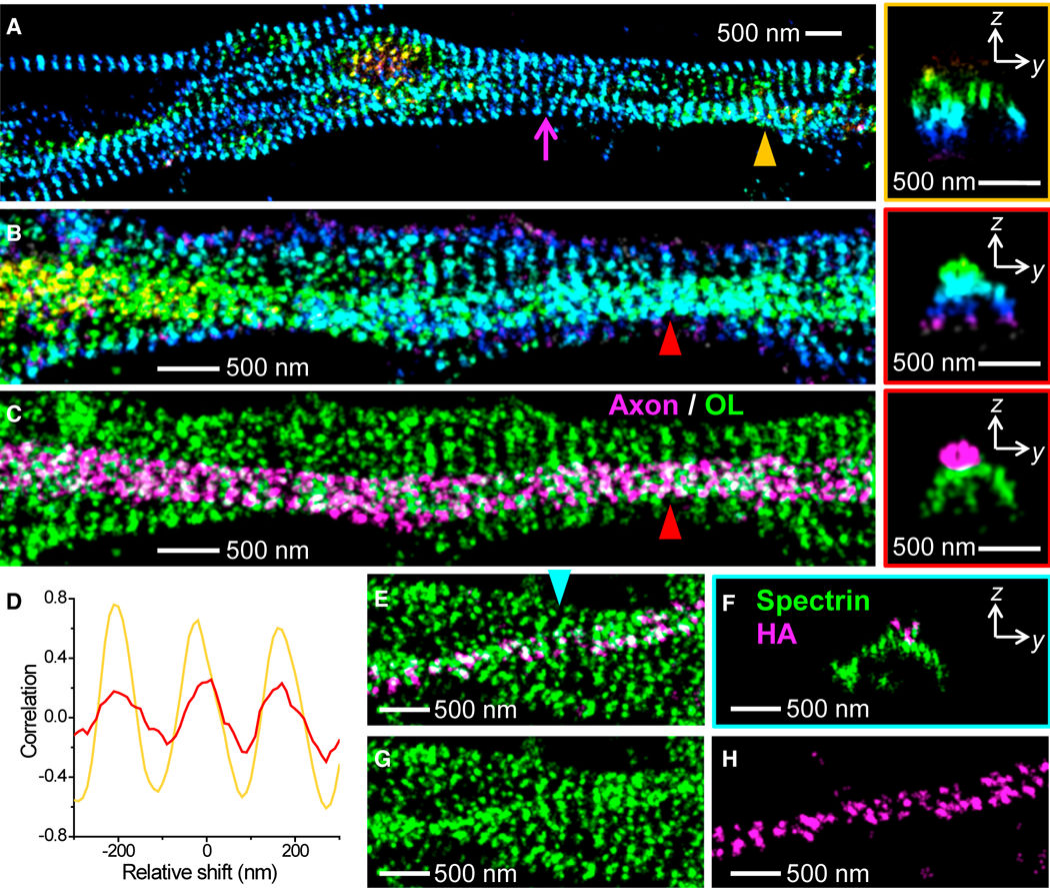
Alignment of the 1D Periodic Membrane Cytoskeletons in Contacting Cells. (A) 3D-STORM image of βII spectrin (C terminus) of contacting axons of primary hippocampal neurons. The magenta arrow points to a lattice mismatch (dislocation). Right: a virtual cross-section in the yz plane for the contacting 1D cytoskeletons pointed to by the orange triangle. (B) 3D-STORM image of βII spectrin (C terminus) of an axon on top of a process of an oligodendrocyte (see MBP staining in Figure S5), in an NSC-derived mixed culture of neurons and glial cells. Right: a virtual cross-section in the yz plane for the contacting 1D cytoskeletons pointed to by the red triangle. (C) Separation of the axon (magenta) and oligodendrocyte (green) membrane cytoskeletons based on the 3D-STORM image in (B). (D) One-dimensional spatial correlations between the spectrin cytoskeletons of two abutting cells for a region surrounding the axon-axon contact site indicated by the orange triangle in (A) (orange curve) and for a region surrounding the axon-oligodendrocyte contact site indicated by the red triangle in (C) (red curve) for varying relative shifts in the horizontal direction. (E) Two-color 3D-STORM image of immunolabeled C terminus of βII spectrin (green, labeled by AF647) and overexpressed HA-tagged C terminus of βII spectrin (magenta, labeled by CF568) in a primary hippocampal culture. (F) A virtual cross-section in the yz plane for the contacting 1D cytoskeletons pointed to by the cyan triangle. (G and H) The two color channels of (E) separated into spectrin (G) and HA-tag (H). See also Figure S5.

We first examined axon-axon interactions in NSC-derived and primary neurons. Remarkably, we found that, when running in parallel, the periodic ring-like spectrin cytoskeletons of abutting axons were often aligned with each other in a one-to-one fashion (Figure 5A; Figures S2 and S5A). 1D spatial correlation for the labeling of two contacting axons showed a high positive value at zero shift as well as repeated peaks corresponding to their mutual ~180 nm spatial periodicity (Figure 5D). Figure 5A further contains a notable case where a mismatch in alignment occurred (magenta arrow). This mismatch appeared to strain and tilt the spectrin rings in the lower axon so that adjacent spectrin rings stayed aligned, analogous to an ‘‘edge dislocation’’ in crystal growth. This forced alignment suggests a marked preference for an aligned periodic membrane cytoskeleton between contacting cells and points to strong binding forces directly associated with the cytoskeletal system.

For axon-glia interactions, Figure 5B captures a case in which an axon grew on top of the thick process of an MBP-positive oligodendrocyte (zoom-out view in Figure S5). Here the excellent height information afforded by 3D-STORM allowed us to separate the cytoskeletal structures of the axon and the oligodendrocyte process (Figure 5C). This revealed that the periodic 1D spectrin cytoskeleton in the axon was mostly aligned with that of the underlying oligodendrocyte process. 1D correlation for the spectrin labeling of the two cells again gave positive maxima at zero and periodic shifts, albeit with reduced amplitudes (Figure 5D). Figure S5 shows more examples: the periodic 1D membrane cytoskeletons of axons and oligodendrocytes were aligned at contact sites.

To further help visualize interactions between different cells, we overexpressed C terminus HA-tagged βII spectrin in a small population of the neurons in a primary hippocampal culture. By immunolabeling the HA tag and the C terminus of βII spectrin with two different dyes, the resultant two-color 3D-STORM images helped to trace down how the HA-tagged spectrin cytoskeleton in an axon interacted with the non-tagged spectrin cytoskeleton in an underlying cell (Figures 5E–5H). This showed that the former is well aligned with the latter at the contact site and that strong interactions between the two apparently strained and tilted the structure of the latter.

### Cell Adhesion Molecules Adopt a Semi-periodic 1D Ultrastructure in Cells of the NSC Lineage

Our results suggest that both axon-axon and axon-glia interactions are modulated by the 1D periodic motif of the membrane cytoskeleton conserved across different cell types. This finding may be explained if cell adhesion molecules (CAMs) (Hortsch, 1996; Maness and Schachner, 2007) responsible for intercellular binding are regulated by the 1D membrane cytoskeletal motif. Indeed, recent work has shown that the CAM neurofascin forms spectrin-actin-regulated periodic structures in the specialized compartments of AISs (D’Este et al., 2015; Leterrier et al., 2015) and nodes of Ranvier (D’Este et al., 2017).

**Figure 6.**
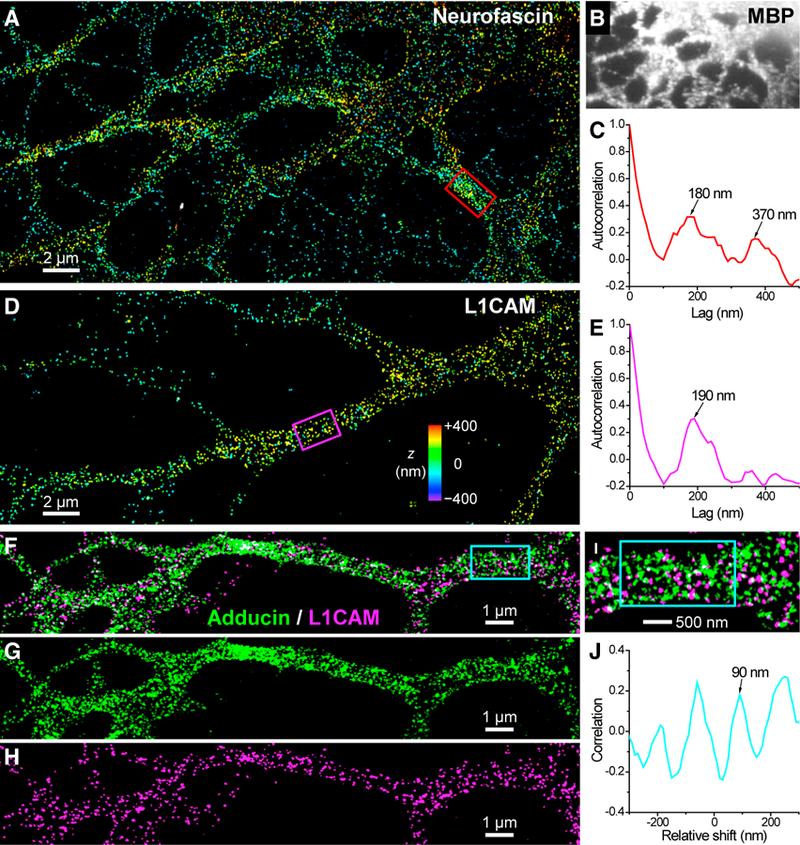
Cell Adhesion Molecules Adopt a Semi-periodic 1D Ultrastructure in NSC- Derived Cells. (A) 3D-STORM image of neurofascin for an NSC-derived oligodendrocyte. (B) MBP staining of the cell. (C) One-dimensional autocorrelation along the red box in (A). (D) 3D-STORM image of L1CAM for an NSC-derived neuron. (E) One-dimensional autocorrelation along the magenta box in (D). (F) Two-color STORM image of adducin (green, labeled by CF568) and L1CAM (magenta, labeled by AF647) in an NSC-derived oligodendrocyte. (G and H) The two color channels of (F) separated into adducin (G) and L1CAM (H). (I) A close-up of (F). (J) One-dimensional spatial correlation between the two color channels for varying relative shifts along the cyan box in (F) and (I). See also Figure S6.

Because neurofascin is also known to be strongly expressed in oligodendrocytes (Collinson et al., 1998; Tait et al., 2000), we examined its ultrastructure in differentiated NSCs. As expected, NSC-derived, MBP-positive oligodendrocytes were characterized by strong neurofascin immunofluorescence (Figures 6A and 6B; Figure S6), whereas astrocytes and neurons showed no labeling except at the AIS (data not shown). 3D-STORM resolved neurofascin in oligodendrocytes as scattered clusters of quasi-periodic patterns (Figure 6A; Figure S6). 1D autocorrelation for a densely labeled process (Figure 6C) showed first and second peaks at 180- and 370-nm lags, respectively, in agreement with the 180- to 190-nm 1D periodicity of the spectrin-actin cytoskeleton we identified in oligodendrocytes (Figures 3D and 3F). These results suggest that, similar to the cases of the AIS (D’Este et al., 2015; Leterrier et al., 2015) and nodes of Ranvier (D’Este et al., 2017), neurofascin in oligodendrocytes is also regulated by the periodic membrane cytoskeleton.

Because neurofascin is only expressed in oligodendrocytes and the specialized compartments of AISs and nodes of Ranvier of neurons, we next asked which other molecules may be responsible for mediating the aligned periodic cytoskeleton we found involving the axon proper (Figure 5). Neurofascin belongs to a major neural CAM group known as the L1 family (Hortsch, 1996; Maness and Schachner, 2007). Within this CAM family, its first member, L1CAM, is widely expressed in the developing nervous system. It plays vital roles in cell-cell interactions and is essential in axon guidance and fasciculation (Engle, 2010; Schäfer and Frotscher, 2012; Wiencken-Barger et al., 2004), as well as in induction of myelination by glial cells (Barbin et al., 2004; Fields, 2015; Wood et al., 1990). Moreover, L1CAM shares consensus ankyrin-binding domains with neurofascin and is functionally coupled with ankyrin and, thus, the spectrin-actin cytoskeleton in premyelinated axons (Davis and Bennett, 1994; Scotland et al., 1998).

We examined the ultrastructure of L1CAM in NSC-derived cells and found that L1CAM adopts a scattered distribution with a moderate local order (Figure 6D; Figure S6). 1D autocorrelation for a process segment showed one peak at 190 nm (Figure 6E), in agreement with the conserved periodic cytoskeletal motif. No notable second peak was observed, indicating lower orderliness in periodicity. This result may be explained if L1CAM occupies a random fraction of the binding sites of ankyrin, while ankyrin itself only exhibits moderate periodicity in neurons (Xu et al., 2013) because of its two possible binding sites close to but not exactly at the centers of spectrin tetramers (Baines, 2010; Bennett and Baines, 2001; Bennett and Gilligan, 1993). In addition, L1CAM may also bind to actin through ezrin-radixin-moesin proteins (Dickson et al., 2002; Maness and Schachner, 2007). Two-color STORM data (Figures 6F–6I) indicated that L1CAM and adducin coexisted on the same periodic cytoskeleton, although the use of a different dye for the second color led to a somewhat reduced image quality for adducin. 1D cross-correlation between the two color channels showed a negative value at zero shift and a maximum at one-half of the 180 nm periodicity (Figure 6J), suggesting an alternating pattern along the 1D periodic cytoskeleton, similar to the case of neurofascin and actin at the AIS (D’Este et al., 2015) and consistent with the notion that L1CAM and neurofascin share consensus ankyrin-binding domains (Davis and Bennett, 1994). Treating the cells with the actin-destabilizing drug latrunculin A led to notably reduced L1CAM labeling and disrupted the structural or- der for both L1CAM and adducin (Figure S6), further indicating that the semi-periodic arrangement of L1CAM depends on the intact spectrin-actin cytoskeleton.

Given the homophilic and heterophilic binding capability of members of the L1 family (Engle, 2010; Fields, 2015; Scha¨ fer and Frotscher, 2012), it is thus possible that the aligned 1D peri- odic spectrin-actin-based cytoskeleton we found at neuron- neuron and neuron-glia interfaces may be linked to the intercel- lular interactions between L1CAM, neurofascin, and possibly other CAM proteins on a 1D periodic cytoskeletal scaffold.

## DISCUSSION

Using 3D-STORM, we unveiled that the NSC has an innate capa- bility to form a spectrin-actin-based periodic membrane cyto- skeleton. Undifferentiated NSCs contained micrometer-sized actin-spectrin-adducin patches of local orderliness, whereas the membrane cytoskeleton of terminally differentiated, mature neurons, astrocytes, and oligodendrocytes is characterized by extensive, long-range 1D periodicity. For developing NSCs in transition to neurons and glial cells, collages of ordered cyto- skeletal strips of 1D periodicity often dominated the flat 2D membranes.

It has been speculated that 2D mem- branes may favor 2D cytoskeleton ar- rangements (Han et al., 2017). Moreover, our recent SRM results (Pan et al., 2018) have shown that the 2D cytoskeletal network of erythrocytes is characterized by an ~80-nm junction-to-junction dis- tance, close to the predicted root-mean- square end-to-end distance of relaxed spectrin tetramers under a thermody- namic equilibrium (Stokke et al., 1985). What gives rise to the highly contrasting, 180- to 190-nm spaced 1D periodic cytoskeleton we observed on the 2D membranes? It has been proposed that parallel bundling may increase the effective rigidity and, thus, the equilib- rium length of spectrin tetramers, a mechanism that has been invoked to model the 180- to 190-nm spaced 1D cytoskeleton in axons (Lai and Cao, 2014). By the same token, in developing NSCs, we found the spectrin-actin cytoskeleton to be arranged into 200- to 300-nm-wide strips within which the 1D periodicity of ~180–190 nm was individually achieved along their micron-scale lengths. This result suggests parallel bundling (fasciculation) of multiple strands of spectrin strings on the 2D membrane during which process molecularly precise alignment is achieved be- tween parallel strings to impart global periodicity. Conceivably, as the NSC differentiates, such a tendency for spectrin strings to bundle on the membrane in an aligned fashion facilitates the ultimate formation of the highly periodic 1D cytoskeleton that un- derlies the entire circumference of the cylindrical processes of neurons, astrocytes, and oligodendrocytes.

To elucidate the potential functions of the highly preserved 1D periodic cytoskeleton across different NSC-derived cell types, we identified remarkable one-to-one cytoskeletal alignments between abutting cells at axon-axon and axon-oligodendrocyte contact sites. Moreover, we found that two L1 family CAMs, neurofascin and L1CAM, adopt quasi-periodic distributions associated with the ~180- to 190-nm periodic 1D cytoskeleton and so serve as possible candidates to drive the cytoskeletal alignment at the nanoscale. L1-family CAMs play critical roles in axon fasciculation (Engle, 2010; Schäfer and Frotscher, 2012; Wiencken-Barger et al., 2004) and are essential to the initial wrapping of glial cells at the axon surface for subsequent myelination (Barbin et al., 2004; Fields, 2015; Wood et al., 1990). Anchoring these key CAMs onto the fixed grids of the 1D periodic membrane cytoskeleton not only provides reliable mechanical coupling but also offers a strategy by which to control CAM density and promote and/or deter adhesion between specific cell types.

Previous results have shown that, during the development of neurons, axons rapidly establish an ordered 1D periodic membrane cytoskeleton from an early stage, whereas dendrites initially lack a periodic cytoskeleton but form local 1D or 2D periodicity at much later times (D’Este et al., 2015; Han et al., 2017; Xu et al., 2013; Zhong et al., 2014). Our discovery that the 1D periodic cytoskeleton assists axon-axon and axon-glia interactions could provide a potential mechanism for cell binding processes to favor axons over dendrites. This effect could be significant because both fasciculation and myelination are functionally important for axons, whereas dendrites tend to avoid analogous intercellular binding. The 1D periodic cytoskeleton thus may serve as a nanoscale molecular ‘‘ruler’’ to select for the desired binding partners and locations. Together, our results indicate that a highly conserved periodic cytoskeletal motif serves as a remarkable scaffold and ruler to mediate the interactions between different cell types of the NSC lineage at the nanoscale.

## EXPERIMENTAL PROCEDURES

### NSC Culture

Rat adult hippocampal NSCs were isolated, cultured, and differentiated as described previously (Peltier et al., 2010a, 2010b). The cells were isolated from 6-week-old female Fisher 344 rats, the most common model in the field, although there are no major differences in the rates of neurogenesis between female and male animals. All animal protocols conformed to the permissions and guidelines at University of California (UC), Berkeley. Cells were plated at ~20,000 cells/cm^2^ on coverslips coated with poly-L-ornithine hydrobromide (Sigma, St. Louis, MO) at 10 μg/mL and natural mouse laminin (Invitrogen, Carlsbad, CA) at 5 μg/mL. Undifferentiated NSCs were maintained in medium consisting of DMEM/nutrient mix F-12 (DMEM/F-12) with 4-(2-hydroxyethyl) piperazine-1-ethanesulfonic acid (HEPES) and L-glutamine (Invitrogen) supplemented with 1% (v/v) N-2 supplement (Invitrogen) and 20 ng/mL basic fibroblast growth factor (FGF-2, PeproTech, Rocky Hill, NJ). Cells were differentiated into a mixed population of neurons, astrocytes, and oligodendrocytes using 1 μM retinoic acid (Biomol, Plymouth Meeting, PA) and 1% (v/v) fetal bovine serum in DMEM/F-12 + N-2 for 5–7 days (for immature cells) or 10–14 days (for mature cells), with medium replacement every 2 days until fixation.

### Primary Neuron and Astrocyte Cultures

Primary rat hippocampal neurons were a kind gift from Prof. Evan Miller’s group or from BrainBits (Springfield, IL) and were plated at ~10,000 cells/cm^2^ on poly-D-lysine-coated coverslips (Neuvitro, GG-12–1.5-PDL) in neuron medium (10 mL B27 and 5 mL GlutaMAX to 500 mL Neurobasal medium [Invitrogen]). Half of the neuron medium was replaced every 3–4 days until fixation at 2–3 weeks. For overexpression of βII spectrin-HA (a gift from Vann Bennett, Addgene plasmid 31070), primary neurons were transfected with 1 μg plasmid per 50,000 cells after 3 days in culture using a calcium phosphate protocol (Jiang and Chen, 2006) and fixed on day 7. Primary hippocampal astrocytes were from BrainBits and plated similarly as neurons but grown in NbAstro medium (BrainBits) and fixed at ~5 days.

### Fixation and Immunolabeling

For experiments aimed at visualizing actin filaments, cells were treated with 0.3% (v/v) glutaraldehyde, 0.25% (v/v) Triton X-100 in cytoskeleton buffer (10 mM 2-(N-morpholino)ethanesulfonic acid [MES] buffer, 150 mM NaCl, 5 mM EGTA, 5 mM glucose, and 5 mM MgCl_2_, adjusted with NaOH to pH 6.1) for 1 min, followed by 2% (v/v) glutaraldehyde in cytoskeleton buffer for 20–30 min (Small et al., 1999; Xu et al., 2012). The sample was treated twice (5 min each) with 0.1% (w/v) NaBH_4_ in PBS and labeled with Alexa Fluor 647 (AF647)-conjugated phalloidin (Invitrogen, A22287, ~0.4 μM). Experiments involving adducin were fixed with 4% paraformaldehyde (PFA) in PBS for 20 min. Experiments with other targets were fixed either with 4% PFA in PBS for 20 min or 3% PFA and 0.1% glutaraldehyde in PBS for 20 min, the latter of which was preceded by 2 3 5 min reduction in 0.1% (w/v) NaBH_4_ in PBS. For immunofluorescence labeling, cells were first blocked with a solution of 3% BSA and 0.1% Triton X-100 in PBS and then stained with corresponding primary and secondary antibodies. The primary antibodies used were as follows: rabbit anti-adducin (Abcam, ab51130, 1:200), mouse anti-βII spectrin (C terminus) (BD Biosciences, 612562, 1:50), mouse anti-nestin conjugated to AF555 (BD Biosciences, 560422, 1:10); mouse anti-nestin (BD Biosciences, 611658, 1:1,000), rabbit anti-GFAP (Abcam, ab7260, 1:1,000), mouse anti-GFAP (Invitrogen, A21282, 1:250), rat anti-myelin basic protein (Abcam, ab7349, 1:100), mouse anti-Tuj (βIII tubulin) (Sigma, SAB4700544, 1:300), chicken anti-Tuj (βIII tubulin) (GeneTex, GTX85469, 1:500); mouse anti-neurofascin (NeuroMab, 73–027, 1:2), mouse anti-L1CAM (Santa Cruz Biotechnology, sc-59868, 1:100), mouse anti-L1CAM (Abcam, ab24345, 1:300), and rabbit anti-HA (Cell Signaling Technology, 3724, 1:1,600). Secondary anti-bodies were from Invitrogen or Jackson ImmunoResearch Laboratories (West Grove, PA) and were AF647- and CF568-labeled for STORM imaging and AF488- and AF555-labeled for cell type markers.

### STORM Super-Resolution Microscopy

3D-STORM (Huang et al., 2008; Rust et al., 2006) was carried out on a home-built setup based on a modified Nikon Eclipse Ti-E inverted fluorescence microscope using an oil immersion objective (Nikon CFI Plan Apochromat λ, 100×, numerical aperture [NA] 1.45), as described previously (Wojcik et al., 2015). Briefly, the sample was mounted with a standard STORM imaging buffer (5% [w/v] glucose, 100 mM cysteamine, 0.8 mg/mL glucose oxidase, and 40 μg/mL catalase in Tris-HCl [pH 7.5]). Lasers at 405, 488, 560, and 647 nm were introduced into the sample through the back focal plane of the objective and shifted toward the edge of the objective to illuminate ~1 μm within the glass-water interface. A strong (~ 2 kW cm ^−2^) excitation laser of 647 nm or 560 nm photoswitched most of the labeled AF647 or CF568 dye molecules into a dark state while also exciting fluorescence from the remaining, sparsely distributed emitting dye molecules for single-molecule localization. A weak (0–1 W cm ^2^) 405-nm laser was used concurrently with the 647-nm or 560-nm laser to reactivate fluorophores into the emitting state so that, at any given instant, only a small, optically resolvable fraction of fluorophores was emitting. A cylindrical lens was used to introduce astigmatism to encode the depth (z) position into the ellipticity of the single-molecule images (Huang et al., 2008). Images were collected at 110 frames per second using an Andor iXon Ultra 897 electron multiplying (EM)-charge-coupled device (CCD) camera for 50,000–120,000 frames per image.

### Data Analysis

Raw STORM single-molecule images were processed into 3D-STORM data and images using previously described methods (Huang et al., 2008; Rust et al., 2006). 1D autocorrelations (for examining the periodicity of a single target) and cross-correlations (for examining the relative positions of two targets) were performed (Xu et al., 2013; Zhong et al., 2014) by shifting the single-molecule localizations in one color channel along one direction (e.g., the lengths of the boxes marked in the figures) by different distances and then calculating the linear correlation coefficient between the shifted and unshifted channels along this direction. Based on this treatment, a correlation value of 1 at zero relative shift would indicate perfect colocalization, whereas −1 would indicate perfect anticorrelation. Positive peaks at non-zero relative shifts would indicate enhanced colocalization at the given shifts; e.g., because of periodic structures.

## Supplementary Material

1

2

## Highlights

- Neural stem cells contain patches of 1D periodic membrane cytoskeletons
- 1D cytoskeletal strips evolve into long-range periodicity in mature neurons and glia
- The conserved 1D periodic cytoskeletal motif is aligned between contacting cells
- This nanoscale alignment is likely due to L1 family CAMs on the periodic cytoskeleton
