# Supplementary material for: The Spectrin-Actin-Based Periodic Cytoskeleton as a Conserved Nanoscale Scaffold and Ruler of the Neural Stem Cell Lineage
Source: Cell Rep. Author manuscript; Available in PMC 2018 Sep 2. (PMC6119594; doi:10.1016/j.celrep.2018.07.005)
Supplement: 1 [file NIHMS1503616-supplement-1.pdf]

Cell Reports, Volume *24*

Supplemental Information

The Spectrin-Actin-Based Periodic Cytoskeleton as a Conserved Nanoscale Scaffold and Ruler of the Neural Stem Cell Lineage

Meghan Hauser, Rui Yan, Wan Li, Nicole A. Repina, David V. Schaffer, and Ke Xu

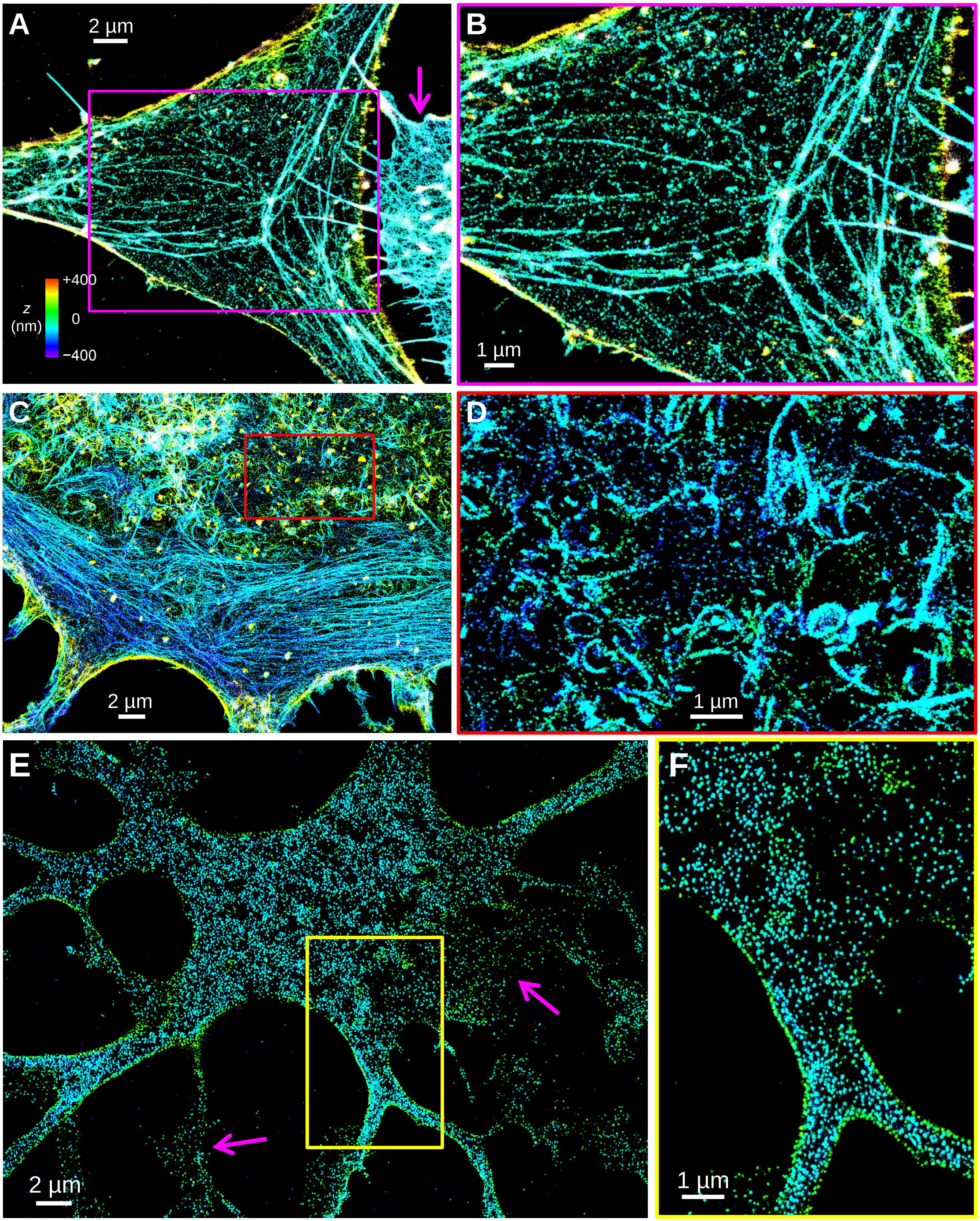

Figure S1. Additional 3D-STORM images of actin and adducin in undifferentiated NSCs. Related to Figures 1 and 2. (A-D) Results of actin. (B) is a zoom-in of the magenta box in (A). (D) is a zoom-in of the red box in (C) for the cytoskeleton at the bottom membrane. (E-F) Results of adducin. (F) is a zoom-in of the yellow box in (E).

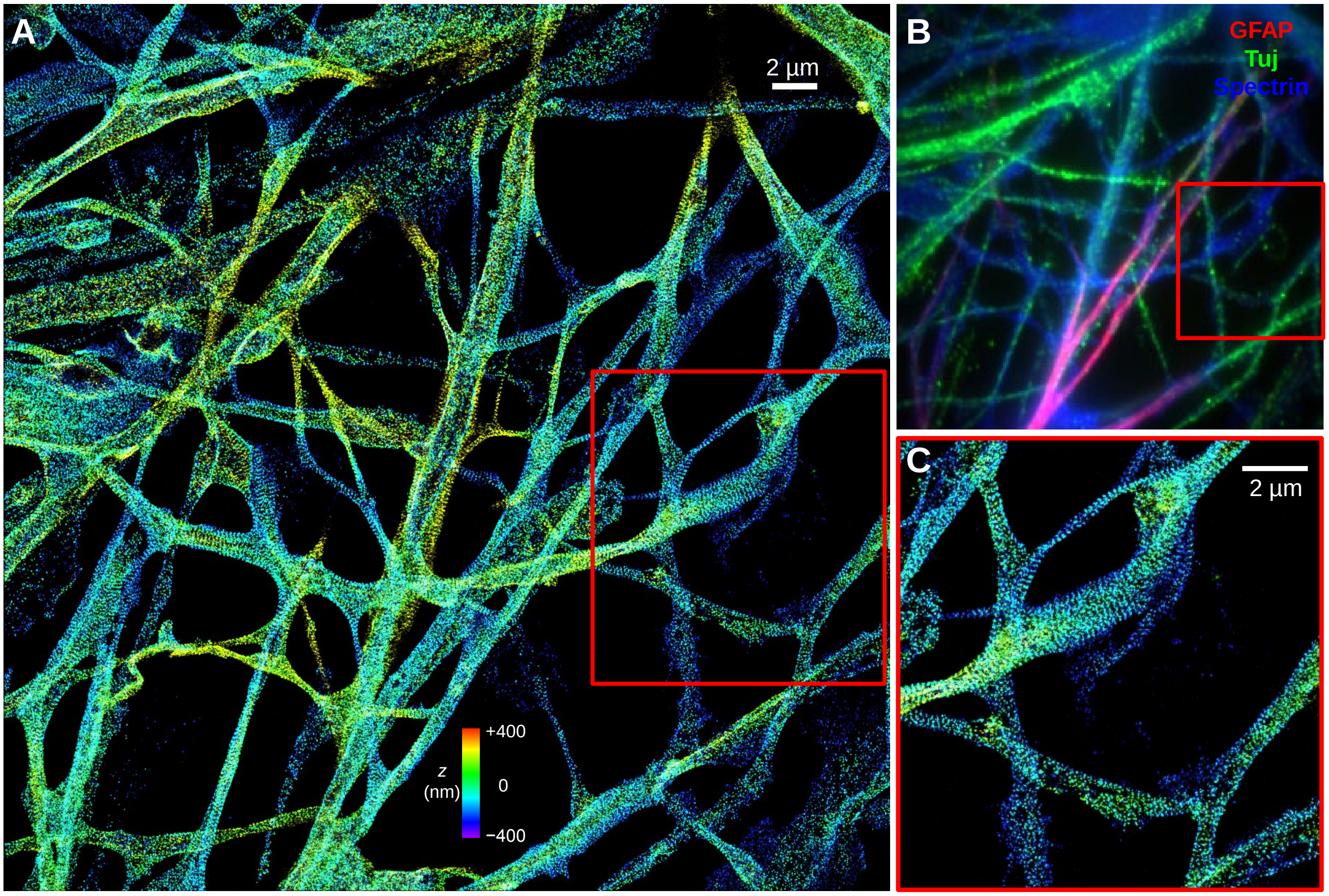

Figure S2. Spectrin in a NSC-derived mixed culture. Related to Figure 3. (A) 3D-STORM image of immunolabeled βII spectrin (C-terminus). (B) Overlaid epifluorescence images of the neuron marker Tuj (green), astrocyte marker GFAP (red), and spectrin (blue), for the same region as (A). (C) Zoom-in of the red box in (A).

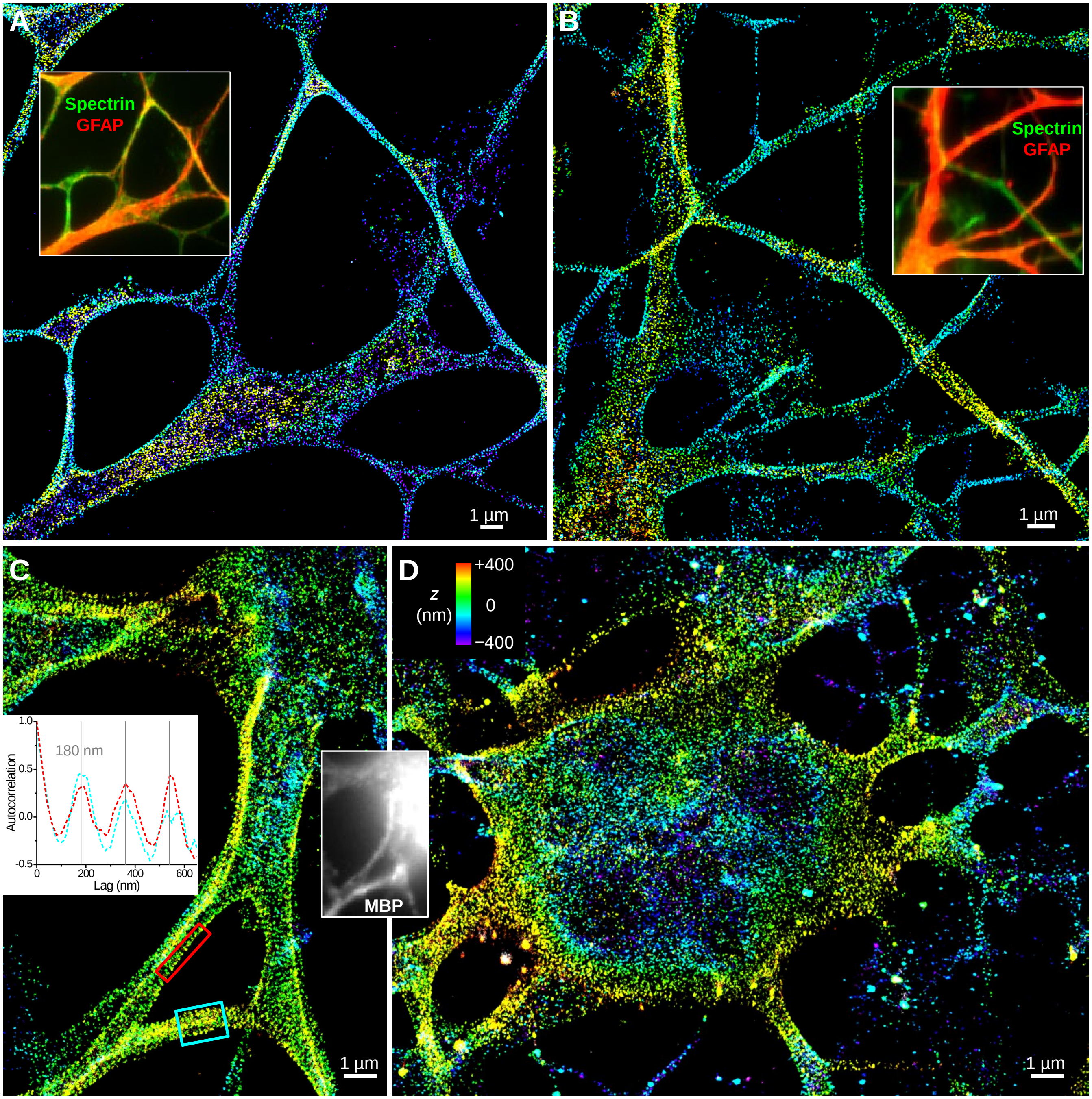

Figure S3. 3D-STORM images of immunolabeled βII spectrin (C-terminus) for NSC-derived astrocytes (A), primary astrocytes (B), and NSC-derived oligodendrocytes (C,D). Related to Figure 3. Insets of (A,B): Immunofluorescence of the astrocyte marker GFAP (red) overlaid with that of spectrin (green). Insets of (C): One-dimensional autocorrelations along the red and cyan boxes, and immunofluorescence of the oligodendrocyte marker MBP.

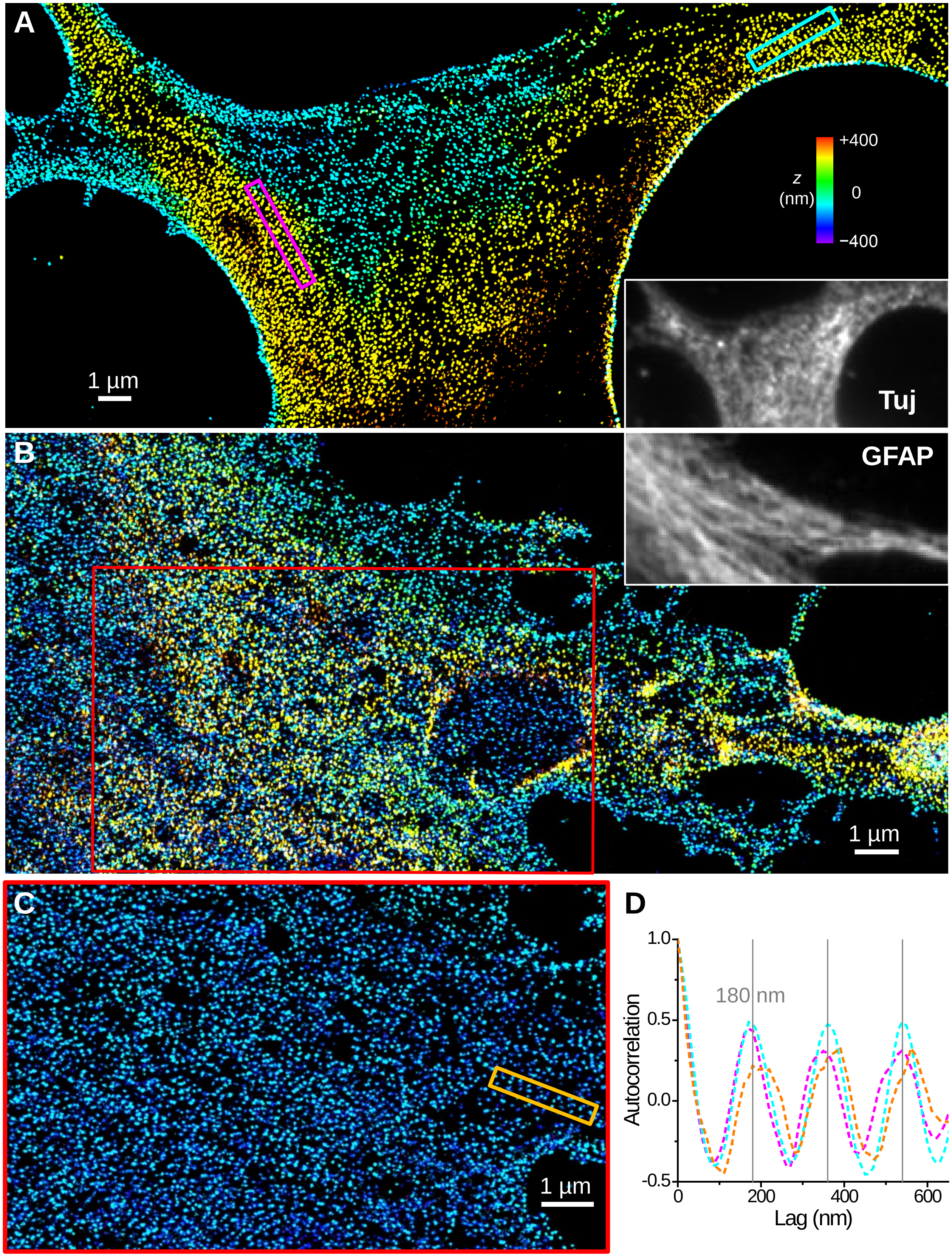

Figure S4. Additional images of 1D periodic motifs on the 2D membranes of developing NSCs. Related to Figure 4. (A) 3D-STORM images of βII spectrin (C-terminus) at the top membrane of an NSC in transition to a neuron. Inset: Immunofluorescence of Tuj. (B) An NSC in transition to an astrocyte. Inset: Immunofluorescence of GFAP. (C) The bottom layer of the red box in (B). (D) One-dimensional autocorrelations along the colored boxes in (A,C).

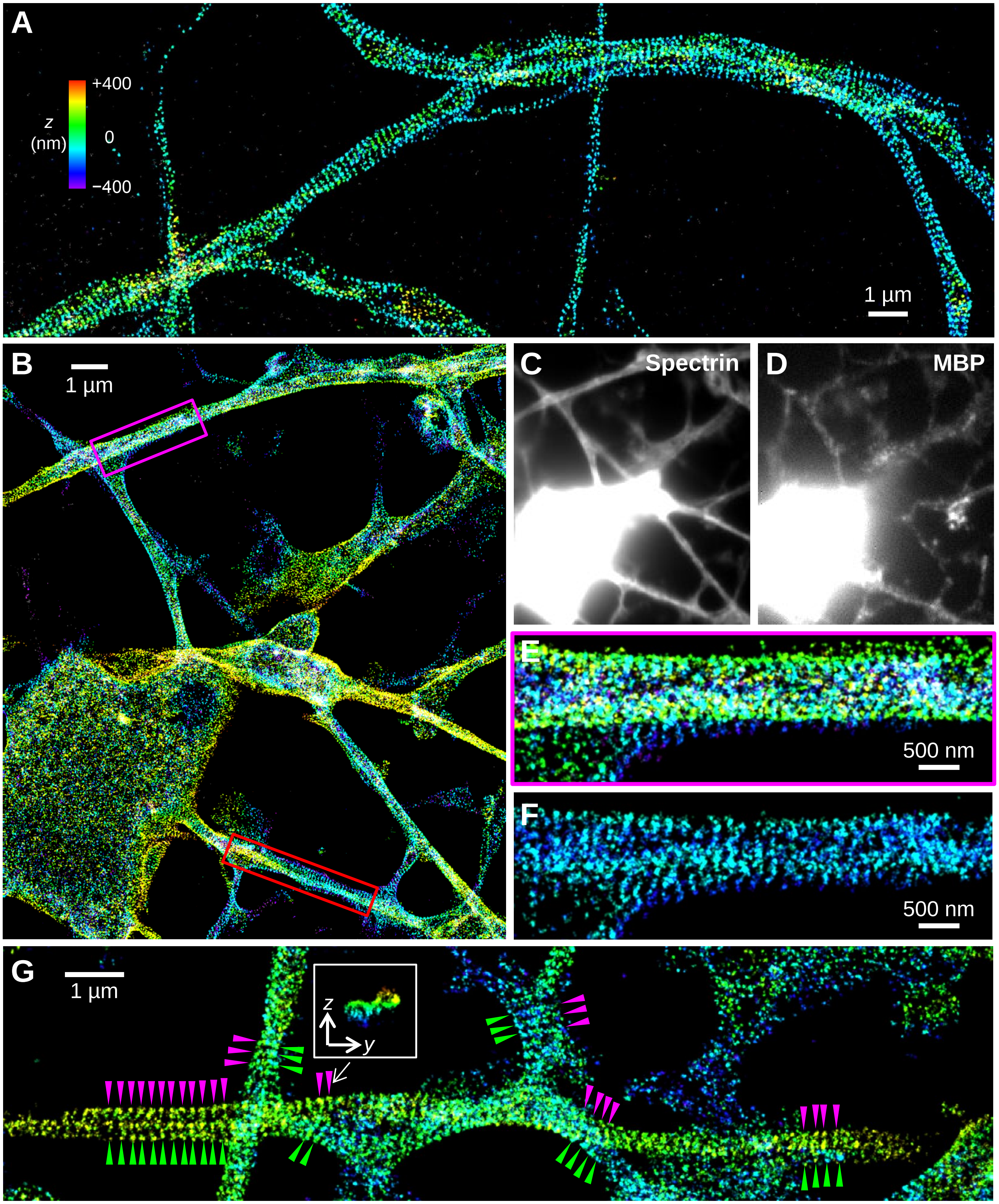

Figure S5. Additional 3D-STORM images of βII spectrin (C-terminus) to show alignment in contacting cells. (A) Axon-axon interactions in primary neurons. Related to Figure 5. (B-G) Axon-oligodendrocyte interactions in an NSC-derived mixed culture. The red box in (B) corresponds to Figure 5B. (C,D) Immunofluorescence of spectrin and the oligodendrocyte marker MBP. (E) Zoom-in of the magenta box in (B). (F) A virtual in-plane slice at the center of the 3D-STORM image, showing structural alignment. (G) Image from another sample. Magenta and green arrowheads point to aligned structures from the axon and oligodendrocyte sides, respectively. Inset: virtual cross-section in the *yz* plane at the position pointed to by the two magenta arrowheads.

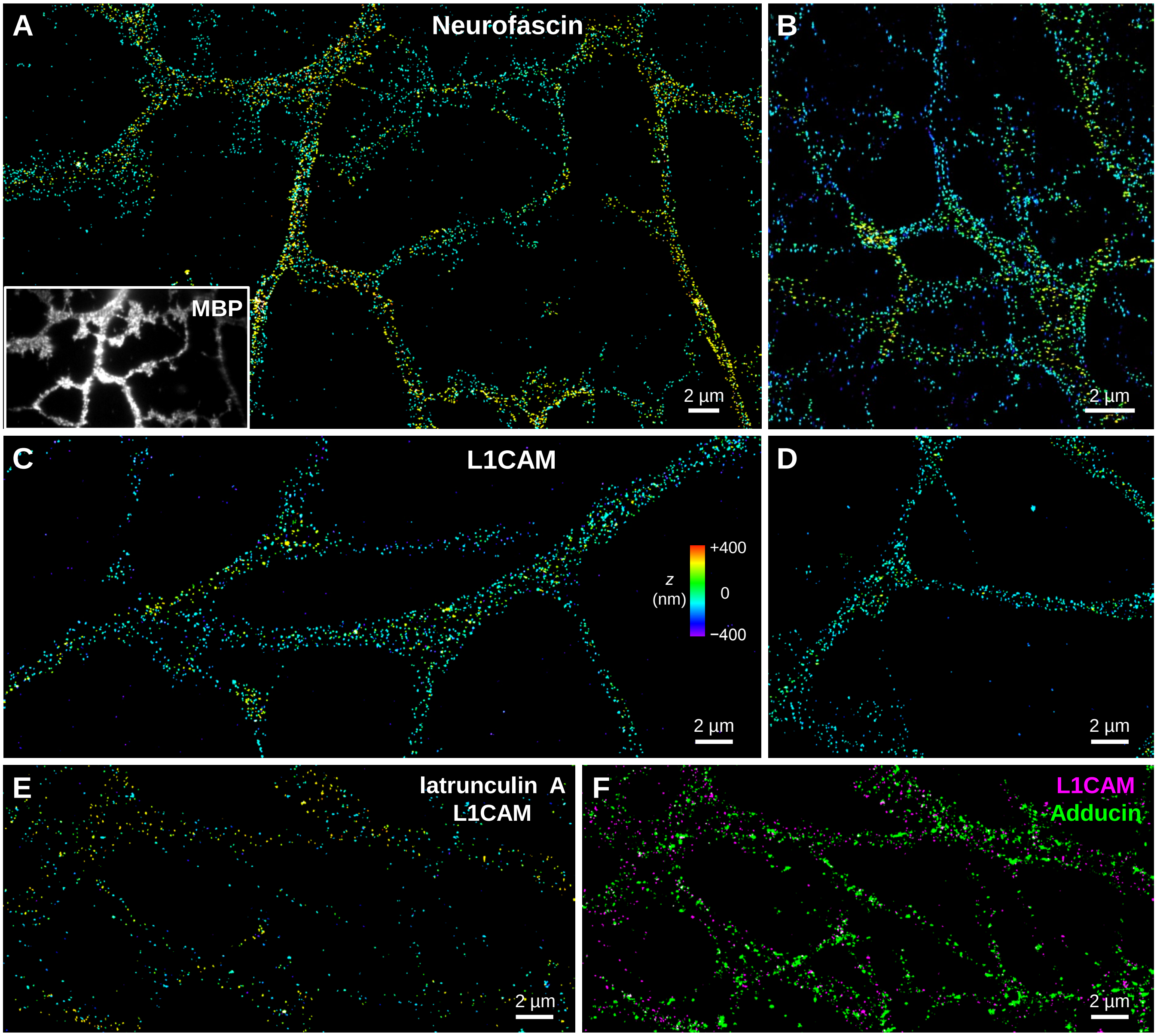

Figure S6. Additional 3D-STORM images of cell adhesion molecules in NSC-derived cells. Related to Figure 6. (A,B) Neurofascin in NSC-derived oligodendrocytes. Inset of (A): Immunofluorescence of MBP. (C,D) L1CAM in NSC-derived neurons. (E) L1CAM in NSC-derived neurons, after treatment with 20 μM latrunculin A for 2 h, showing markedly lower density. (F) The same image recolored to magenta and overlaid with STORM result of adducin (green). The cytoskeleton appears noticeably disrupted after drug treatment, suggesting a mechanism by which L1CAM number could also be altered.
